# Use of Hepatitis C Virus Antibody-Positive Donors in Kidney Transplantation

**DOI:** 10.7759/cureus.51849

**Published:** 2024-01-08

**Authors:** Sofia Ventura, Cátia Figueiredo, Círia Sousa, Manuela Almeida, La Salete Martins

**Affiliations:** 1 Nephrology, Hospital do Divino Espírito Santo, EPER, Ponta Delgada, PRT; 2 Nephrology, Centro Hospitalar do Médio Tejo, Torres Novas, PRT; 3 Nephrology, Centro Hospitalar de Trás-os-Montes e Alto Douro, Vila Real, PRT; 4 Nephrology, Centro Hospitalar Universitário do Porto, Porto, PRT

**Keywords:** immunosuppresion, post-renal transplant, viral serology, hepatitis c (hcv) infection, • kidney transplantation

## Abstract

Background

The use of kidney donors with hepatitis C virus (HCV) has been arising as a possibility to increase the donor pool. It encompasses both the use of donors with positive and negative viremia, particularly since the advent of direct antiviral agents that produce sustained virologic response.

Methodology

We conducted a retrospective observational study to describe the experience of our transplantation center in the use of HCV antibody-positive (HCV-Ab+) kidneys.

Results

We performed five transplants with HCV-Ab+ donors. The median age of kidney recipients was 63 (interquartile range (IQR) = 54-71) years, and 60% (n = 3) were males. Two recipients received a second transplant. The median dialysis vintage was 1,414 (IQR = 1,103-2,806) days. The induction immunosuppression protocol was basiliximab in most patients (60%, n = 3), and all received maintenance immunosuppression with tacrolimus, mycophenolate mofetil, and prednisolone. One of the recipients had a personal history of cured HCV infection. Seroconversion occurred in half of the remaining patients, which was sustained during the follow-up. None of the patients developed HCV viremia. At the end of follow-up, mean creatinine and proteinuria were 1.45 ± 1.12 mg/dL and 0.099 ± 0.045 g/g, respectively. We did not observe any rejection episodes, need for dialysis, or recipient’s death.

Conclusions

Our work aligns with the current literature that advocates that the use of these donors is safe and cost-effective and can be an effective strategy for expanding the donor pool and augmenting the transplantation volume. Seroconversion is a known risk whose mechanisms are not entirely understood, although it does not appear to be related to a higher transmission risk.

## Introduction

Kidney transplantation is the preferred treatment for end-stage kidney disease (ESKD), extending patients’ life expectancy and being more cost-effective compared to long-term dialysis [1]. However, there is a continued organ shortage for transplantation and a significant disparity between organ supply and demand, especially for patients with blood types O and B, resulting in increased waiting times and high mortality rates among patients on the waiting list [2-4]. This has stimulated the use of organs from increased‐risk donors, namely, in the presence of a higher risk of infection transmission [2-4]. One of these strategies, which has been of growing interest in recent years, is the use of donors with hepatitis C virus (HCV) infection.

At the time of transplantation, all potential kidney donors are submitted to HCV nucleic acid test (NAT), performed by polymerase chain reaction (PCR), regardless of previous serology [5]. This practice is especially relevant in HCV‐seropositive (HCV-Ab+) donors, confirming the presence or absence of active infection with high sensitivity and specificity, respectively, subdividing patients into HCV-Ab+/NAT+ (positive viremia) and Ab+/NAT- (negative viremia).

It is established that kidneys from HCV-Ab+/NAT+ donors are offered to recipients who are also HCV-Ab+/NAT+ [4,6-8]. This practice has led to positive outcomes and good safety and efficacy profiles, allowing for the use of excellent quality organs as these donors tend to be younger than the general donor population. Additionally, it leads to decreased waitlist times and increased access to transplantation, as well as a potential decrease in long-term morbidity and mortality for this population [4,6-9].

Because of these positive outcomes, there has been increased interest in transplanting kidneys from HCV-positive donors into uninfected recipients [8]. This approach has become possible after the advent of direct-acting antivirals (DAAs), which have significantly changed the treatment of HCV infection, allowing for cure rates exceeding 95% through a well‐tolerated treatment with short duration [2,3,8], as compared to interferon, which led to low sustained virologic response (SVR) and was associated with unfavorable side effects, including risk of kidney injury and graft loss [3,4,10,11].

The actual risk of transmission in the use of HCV-Ab+/NAT+ kidneys in recipients without HCV infection remains to be accurately defined. Molnar et al. [6] showed positive results in this setting, and, although there was an occurrence of disease transmission, all recipients showed only transient viremia and maintained excellent short-term allograft function without patient or graft losses. Regarding the use of HCV-Ab+/NAT- donors, only small single-center studies have reported that the HCV transmission risk appears to be negligible [2,12,13].

Discussions with the patient about the pros and cons of accepting a kidney from an HCV-Ab+ donor should include their time under dialysis, regional waitlist times, urgency of transplantation, and how sensitized the patient is [8]. Due to the risk of disease transmission, any patient considered for an HCV-positive organ should be evaluated by a hepatologist to ensure that they will be a candidate for DAA treatment or a liver transplant in the case of progressive liver injury [8].

In Portugal, the transplantation of HCV-Ab+ kidneys has been possible since 2018, provided the HCV-Ab+/NAT+ organs are allocated to recipients with HCV infection, regardless of the serologic subtype; the HCV-Ab+/NAT- organs are allocated to recipients without HCV infection. If NAT results are unavailable, the case should be managed as if it is positive. Explicit informed consent must be obtained from the recipients before transplantation.

## Materials and methods

The goal of this study was to describe the experience of a Portuguese transplantation center in the utilization of kidneys from HCV-Ab+/NAT- donors in negative recipients.

We conducted a retrospective observational study including all patients transplanted with kidneys from HCV-Ab+/NAT- donors between October 2018 and September 2022. There were no exclusion criteria.

We reviewed the patient’s electronic medical records and collected data on their demographics, clinical and analytical variables, and transplant outcomes. Demographic variables included gender and age at the time of transplantation. Clinical variables included ESKD etiology, pre-transplant dialysis modality and vintage, type of donation (deceased vs. living donor), induction immunosuppression and immunomodulation, number of human leukocyte antibody (HLA) mismatches, and presence or absence of donor-specific antibodies (DSAs). Analytical variables included serum creatinine (in mg/dL) and proteinuria (in g/g) collected post-transplant (at three and six months, and one, two, and three years), as well as HCV-Ab status and HCV viremia. We also accessed post-transplant kidney variables, namely, graft and patient survival at the end of follow-up and the occurrence of any rejection episodes.

## Results

Between October 2018 and September 2022, we performed five transplants from HCV-Ab+/NAT- donors, most of which (80%, n = 4) were from deceased donors with expanded criteria and one patient from a related living donor. Most recipients (60%, n = 3) were males, and the median recipient age at the time of transplantation was 63 years (interquartile range (IQR) = 54-71). Two patients (40%) had ESKD as a consequence of diabetic nephropathy, two (40%) had chronic interstitial nephropathy, and one (20%) had nephropathy of undetermined etiology.

The median time of ESKD treatment before transplantation was 1,414 days (IQR = 1,103-2,806), and most patients were under hemodialysis (80%, n = 4). Two (40%) patients received a second transplant. The clinical characteristics of recipients and donors are summarized in Table 1.

**Table 1:** Clinical characteristics of recipients and donors. ESKD: end-stage kidney disease; IQR: interquartile range

Recipient’s characteristics (n = 5)	
Age at time of transplantation (years), median (IQR)	63 (54–71)
Gender – male, n (%)	3 (60)
ESKD time before transplantation (days), median (IQR)	1,414 (1,103–2,806)
ESKD therapy – hemodialysis, n (%)	4 (80)
Donor’s characteristics (n = 4)	
Age at time of donation (years), median (IQR)	56.5 (54.25–58.5)
Gender – male, n (%)	2 (50)
Type of donation – deceased donor, n (%)	3 (75)

Induction immunosuppression and immunomodulation therapies are summarized in Table 2. Concerning immunological characteristics, the median number of HLA mismatches was 3 (minimum = 1, maximum = 4). One patient had positive DSA, corresponding to the one that received higher immunosuppression. All patients showed a negative crossmatch by flow cytometry. Most patients (60%, n = 3) received non-depleting induction therapy with basiliximab. Additionally, we used immunomodulation with rituximab in 60% and human intravenous immunoglobulin (IVIg) in 20%. We highlight that the patient who received a living donor organ was also subjected to plasma exchange therapy as part of the protocol for an AB0-incompatible transplant.

**Table 2:** Immunosuppression regimens and other transplant-related variables.

Transplant-related variables	
Induction immunosuppression protocol (+ mycophenolate mofetil and methylprednisolone)	
Basiliximab, n (%)	3 (60)
Anti-thymocyte immunoglobulin, n (%)	2 (40)
Rituximab usage, n (%)	3 (60)
Intravenous immunoglobulin usage, n (%)	1 (20)
HLA mismatch number, median (minimum-maximum)	3 (1-4)
Donor-specific antibodies presence, n (%)	1 (20)
Negative crossmatch, n (%)	5 (100)

The median follow-up time after transplantation was 706 days (IQR = 706-1,412), with two patients having follow-up time greater than two years. Mean creatinine (±SD) at three and six months were 1.37 ± 0.28 mg/dL and 1.49 ± 0.41 mg/dL, respectively (reference value = 0.7-1.2 mg/dL). Mean proteinuria (±SD) at six months after transplantation was 0.192 ± 0.055 g/g in a spot urine sample (reference value = 0.015-0.068 g/g). At three years post-transplant, mean creatinine levels were 1.45 ± 1.12 mg/dL, and mean proteinuria was 0.099 ± 0.045 g/g. The clinical outcomes of all recipients are summarized in Table 3.

**Table 3:** Analytical and clinical outcomes in the transplant recipients. *: Decrease in the sample to two patients. Reference values for creatinine: 0.7-1.2 mg/dL. Reference values for proteinuria: 0.015-0.068 g/g.

Clinical outcomes in the transplant recipients	
Creatinine (mg/dL), mean ± SD	
At 3 months	1.37 ± 0.28
At 6 months	1.49 ± 0.41
At 1 year	1.35 ± 0.31
At 2 years*	1.27 ± 0.35
At 3 years*	1.45 ± 1.12
Proteinuria (g/g), mean ± SD	
At 1 year	0.192 ± 0.055
At 2 years*	0.099 ± 0.028
At 3 years*	0.099 ± 0.045
Rejection episodes during follow-up, n (%)	0 (0)
Graft survival at the end of follow-up, n (%)	5 (100)
Recipient survival at the end of follow-up, n (%)	5 (100)

We did not observe any rejection episodes, and patient and graft survival were 100% at the end of the follow-up period.

One of the transplant recipients had a previous history of HCV infection and was submitted to DAA treatment approximately three years pre-transplantation, achieving SVR. Half of the remaining patients (n = 2) seroconverted during the follow-up period and became HCV-Ab+ at one month and two years of follow-up. This seroconversion was persistent during the remaining follow-up period. All recipients remained aviremic (Table 4).

**Table 4:** Kidney recipients’ HCV serology and viremia after transplantation. *: Patient with a prior history of HCV infection. HCV: hepatitis C virus; N/A: not applicable

Patient ID	Pre-transplant		1 month after transplant		3 months after transplant		6 months after transplant		12 months after transplant		18 months after transplant		24 months after transplant	
	Antibody	Viremia	Antibody	Viremia	Antibody	Viremia	Antibody	Viremia	Antibody	Viremia	Antibody	Viremia	Antibody	Viremia
1	Negative	Negative	Negative	Negative	Negative	Negative	Negative	Negative	Negative	Negative	Negative	Negative	Positive	Negative
2	Negative	Negative	Negative	Negative	Negative	Negative	Negative	Negative	Negative	Negative	Negative	Negative	Negative	Negative
3	Positive*	Negative	N/A	Negative	N/A	Negative	N/A	Negative	N/A	Negative	N/A	Negative	-	-
4	Negative	Negative	Positive	Negative	Positive	Negative	Positive	Negative	Positive	Negative	Positive	Negative	-	-
5	Negative	Negative	Negative	Negative	Negative	Negative	Negative	Negative	Negative	Negative	-	-	-	-

## Discussion

We highlight the excellent safety profile of using kidneys from anti-HCV+ donors in our cohort. We observed a transmission rate of 0%, absence of rejection episodes, and 100% patient and graft survival rates at the end of follow-up time, even when using a patient with previous HCV infection and SVR. This is consistent with other published studies [12,14,15] which reported zero HCV transmissions in small groups of transplant recipients among 4, 21, and 22 patients, respectively, studied retrospectively with follow-up periods ranging between 12 and 50.7 months. Nonetheless, it is important to report that a prospective study by Dao and colleagues [2], which included 52 patients, showed a disease transmission rate of 1.9%, corresponding to one patient who developed HCV viremia. The possible explanations were transient low‐level viremia, the presence of occult infection in the donor tissues, possible false‐negative initial HCV-NAT test, and eclipse period infection.

We observed the occurrence of seroconversion in half of our study population, consistent with the seroconversion rates in the existing literature, which can go up to 42-44% [15,16]. This phenomenon is known to arise only in a subset of patients, and in most cases, it is persistent, although the risk factors for its occurrence have not been fully explained [2,15]. It has been postulated that passive transfer of donor HCV antibodies may occur in recipients regardless of NAT status [17] or that the recipients react to antigens in the renal tissue, for example, HCV‐NS3 [15].

In summary, although the transplantation of HCV-Ab+/NAT- kidneys to HCV-negative patients seems to provide a good safety profile, it is relevant to highlight that there may be a shallow risk of transmission, rather than a zero risk. This is crucial for clinicians to give their patients the most accurate information pre-transplantation, with efficient informed consent, and to implement clinical protocols to access HCV antibody and viremia positiveness in kidney recipients who are at the highest risk of disease transmission.

It is necessary to discuss the limitations and the positive aspects of our study. The limitations include its retrospective nature, the small sample size, and the short follow-up duration. The positive aspects are that this study represents the largest cohort of Portuguese patients with HCV-Ab+ donors, to our knowledge, and with the longest follow-up period, considering that transplantation from HCV-Ab+/NAT- donors to patients without HCV infection has only been possible in Portugal since 2018. Moreover, this kind of transplantation is still not a common practice, and we consider that it is crucial to publish the existing data and review the literature on this subject to amplify this procedure in clinical practice.

## Conclusions

The use of HCV-Ab+/NAT- kidneys in transplantation appears to be safe, even when giving these organs to HCV-negative patients. The expansion of its use can be an effective strategy for expanding the donor pool and increasing the transplantation volume. Informed consent must be taken from the patients as there may be a shallow risk of virus transmission. It is important to acquire more information on this topic to increase knowledge and, therefore, to amplify its use. Our study, although using a small patient sample, reinforces the safety and the good outcomes that can be obtained through this technique. More studies are required, especially randomized controlled trials.
